# The influence of season on glutamate and GABA levels in the healthy human brain investigated by magnetic resonance spectroscopy imaging

**DOI:** 10.1002/hbm.26236

**Published:** 2023-02-25

**Authors:** B. Spurny‐Dworak, M. B. Reed, P. Handschuh, T. Vanicek, M. Spies, W. Bogner, R. Lanzenberger

**Affiliations:** ^1^ Department of Psychiatry and Psychotherapy, Comprehensive Center for Clinical Neurosciences and Mental Health (C3NMH) Medical University of Vienna Vienna Austria; ^2^ Department of Biomedical Imaging and Image‐Guided Therapy, High Field MR Centre Medical University of Vienna Vienna Austria

**Keywords:** GABA, glutamate, light, MRS, season

## Abstract

Seasonal changes in neurotransmitter systems have been demonstrated in imaging studies and are especially noticeable in diseased states such as seasonal affective disorder (SAD). These modulatory neurotransmitters, such as serotonin, are influencing glutamatergic and GABAergic neurotransmission. Furthermore, central components of the circadian pacemaker are regulated by GABA (the suprachiasmatic nucleus) or glutamate (e.g., the retinohypothalamic tract). Therefore, we explored seasonal differences in the GABAergic and glutamatergic system in 159 healthy individuals using magnetic resonance spectroscopy imaging with a GABA‐edited 3D‐MEGA‐LASER sequence at 3T. We quantified GABA+/tCr, GABA+/Glx, and Glx/tCr ratios (GABA+, GABA+ macromolecules; Glx, glutamate + glutamine; tCr, total creatine) in five different subcortical brain regions. Differences between time periods throughout the year, seasonal patterns, and stationarity were tested using ANCOVA models, curve fitting approaches, and unit root and stationarity tests, respectively. Finally, Spearman correlation analyses between neurotransmitter ratios within each brain region and cumulated daylight and global radiation were performed. No seasonal or monthly differences, seasonal patterns, nor significant correlations could be shown in any region or ratio. Unit root and stationarity tests showed stable patterns of GABA+/tCr, GABA+/Glx, and Glx/tCr levels throughout the year, except for hippocampal Glx/tCr. Our results indicate that neurotransmitter levels of glutamate and GABA in healthy individuals are stable throughout the year. Hence, despite the important correction for age and gender in the analyses of MRS derived GABA and glutamate, a correction for seasonality in future studies does not seem necessary. Future investigations in SAD and other psychiatric patients will be of high interest.

## Graphical abstract

Here we investigated seasonal influences on baseline concentrations of GABA and glutamate across five different subcortical brain regions of healthy subjects. Moreover, associations of light and radiation with derived neurotransmitter measures were tested. Hence, we found stable patterns of neurotransmitter levels throughout the year.

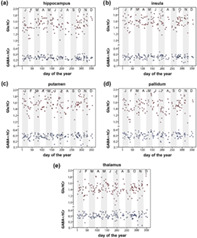

## 1 INTRODUCTION

Environmental conditions can change drastically over the course of the year and thus require an adequate physiological response from the human body. The brain especially adapts to these variations and in turn is influenced by seasonality. Furthermore, seasonal changes are reflected in behavioral and mood shifts (Kasper et al., 1989), impact on cognitive function (Meyer et al., 2016), and slight variations in brain volume (Book et al., 2021). Physiological adaptions in terms of altered neurotransmission were previously described in the dopaminergic and serotonergic system when dopamine synthesis was reported to follow seasonal patterns in health (Eisenberg et al., 2010) and disease (Kaasinen et al., 2012). Moreover, phenomena like photoperiod‐induced neurotransmitter plasticity (Porcu et al., 2022) or altered numbers of midbrain dopaminergic neurons in summer compared to winter were reported (Aumann et al., 2016). Distinct seasonal variations in the serotonergic neurotransmitter system have often described in pathological conditions such as seasonal affective disorder (SAD) (Daut & Fonken, 2019). As reviewed by Partonen and Lonnqvist (1998), SAD is characterized by the occurrence or a significant increase of depressive symptoms, mainly in autumn and winter and is believed to be linked to light exposure (Partonen & Lonnqvist, 1998). Several seasonal variations in the serotonergic system could be shown by our group in serotonin transporter (SERT) levels (Mc Mahon et al., 2016; Praschak‐Rieder et al., 2008), monoamine oxidase A (Spies et al., 2018), or the serotonergic receptor 1A (Spindelegger et al., 2012). In addition, bright light therapy showed properties to affect concentrations of SERT and monoamine oxidase A, suggesting modulatory effects of light exposure on the serotonergic system (Spies et al., 2018; Willeit et al., 2008) and is therefore considered an effective treatment options (Pjrek et al., 2020). Moreover, in animal studies a link between the serotonergic and GABAergic system could be provided when hippocampal concentrations of serotonin and GABA were shown to follow seasonal rhythms in rats (Li et al., 2020).

Since major depressive disorder (MDD) is associated with changes in monoamines and other neurotransmitter systems, including GABA (Kalueff & Nutt, 2007; Schur et al., 2016) and glutamate (Sanacora et al., 2012). Therefore, seasonal adaptions within these systems are of high interest for both clinical reasons as well as methodological aspects. Interestingly, GABA and glutamate are not only the main inhibitory and excitatory neurotransmitter of the human brain, respectively, but are also the main neurotransmitters innervating the key brain structures for seasonal rhythms in humans. The retinohypothalamic tract (RHT) projecting from the retina to the suprachiasmatic nucleus (SCN), located in the hypothalamus, provides the anatomical basis for the light‐dependent regulation of the circadian rhythm in mammals (Hannibal, 2021; Mendoza, 2017). While the RHT is under glutamatergic control (Ebling, 1996; Hannibal, 2006), the SCN is mainly innervated by GABAergic neurons (Albers et al., 2017; Ono et al., 2021). Glutamate, the primary mediator in the RHT of light signaling the circadian rhythm is involved in both circadian and seasonal activities across species by inducing light‐dependent phase shifts via NMDA and AMPA receptors in rodents (Ebling, 1996). Moreover, several studies highlighted the importance of the GABAergic system for circadian and seasonal processes within this and related brain regions. To ensure adequate adaptions of brain function along the day and year, extracellular levels of GABA in SCN are meticulously controlled by complex interactions of synaptic and nonsynaptic release mechanisms as well as transport and synthesis (Albers et al., 2017). On a cellular level GABA‐mediated coupling of circadian clock neurons of the SCN, by modulation of intracellular chloride concentrations, was reported to encode seasonal time periods (Myung et al., 2015). Vice versa it was demonstrated that differences in light exposure can influence GABAergic neurotransmission in mice, showing shifts in GABAergic activities from inhibition towards excitation, when photoperiods were switched to long‐day photoperiods, emphasizing the influence in environmental conditions on neurotransmitter systems (Farajnia et al., 2014). On a larger‐scale GABA‐mediated cortical inhibition was shown to follow circadian patterns, revealing changes in GABA levels beyond the RHT (Lang et al., 2011). Hence, seasonal rhythms in the human are not only controlled by GABA and glutamate in key brain regions, but are thought to influence a variety of other brain regions (Lang et al., 2011). Thus, seasonal variations in the GABAergic and glutamatergic system, mainly reported in animal models, need to be investigated across different brain regions in human research to shed light on disease‐linked alterations in neurotransmitter systems. We therefore quantified baseline concentrations of GABA+ (a combination of GABA and macromolecules) and Glx (glutamate+glutamine) ratios to total creatine (tCr) in five subcortical brain regions (hippocampus, insula, putamen, pallidum, and thalamus) of healthy individuals, collected at different dates throughout the year using magnetic resonance spectroscopy imaging (MRSI). Regions of interest were either included in these analyses based on their role in circadian regulation of cerebral function [e.g., the hippocampus (Ruby et al., 2008; Snider et al., 2018) or thalamus (Alamilla et al., 2015)] or their role in the pathophysiology of depression, especially SAD [e.g., insula (Pastrnak et al., 2021; Schnellbacher et al., 2022), putamen (Sacchet et al., 2017; Talati et al., 2022) or pallidum (Norgaard et al., 2017)]. Beside the importance of seasonal differences of the main inhibitory and excitatory neurotransmitter system in healthy individuals, a potential need for correction of MRS derived measures of GABA+ and Glx is of interest.

## 2 METHODS

In the course of this analyses, we aimed to investigate seasonal patterns in neurotransmitter ratios of healthy individuals, derived from MRSI. To this end, baseline measurements of four different studies (Silberbauer et al., 2020; Spurny et al., 2021; Spurny‐Dworak et al., 2022) using the same MRSI sequence and parameters, were pooled. All studies were approved by the ethics committee of the Medical University of Vienna (EK 1739/2012, 1104/2015, 2169/2016, 2170/2016, 1410/2020) and were performed in accordance with the Declaration of Helsinki 1964. Participants gave written consent and received financial reimbursement for their participation.

### 2.1 Study cohort

Baseline measurements from a total of 159 healthy participants (79, female, mean age ± SD = 25.4 ± 5.3 years, ranging from 18 to 50 years) were included into our analyses. All participants were free from internal, neurological, or psychiatric disorders. Exclusion criteria included current or former substance abuse, lifetime use of SSRIs or related psychotropic agents, smoking, first‐degree relatives with a history of psychiatric illness, or any contraindications for MRI. Urine drug and pregnancy tests (for women) were performed prior to each MRI session.

### 2.2 MRS measurements and data analysis

All MRI measurements were performed on a 3 Tesla MR Magnetom Prisma system (Siemens Medical, Erlangen, Germany) installed at the High‐field MR Center, Department of Biomedical Imaging and Image‐guided Therapy, Medical University of Vienna, using a 64‐channel head coil. Structural T1‐weighted images (TE = 1800 ms, TR = 2.37 ms, 208 slices, 288 × 288 matrix size, voxel size 1.15 × 1.15 × 0.85 mm^3^) were acquired prior to each MRSI scan for accurate volume of interest (VOI) placement and mask extraction for the region‐of‐interest (ROI)‐based quantification. MRSI measurements were conducted using a 3D GABA‐edited MEGA‐LASER MRSI sequence described in (Bogner, Gagoski, et al., 2014) including real‐time correction for rigid body motion and center frequency changes (Bogner, Hess, et al., 2014) with a TE of 68 ms. MEGA‐editing pulses utilizing 60 Hz Gaussian pulses of 14.8 ms duration were set to 1.9 ppm during EDIT‐ON acquisition. The VOI was placed parallel to the anterior commissure–posterior commissure line to cover all five regions of interest bilaterally, with a VOI = 80 (l‐r) × 90 (a‐p) × 80 (s‐i) mm^3^ and a field of view (FOV) = 160 × 160 × 160 mm^3^ (see Figure S1, Supporting Information). The acquired matrix size of 10 × 10 × 10 (i.e., ~4 cm^3^ nominal voxel size) was interpolated to a 16 × 16 × 16 matrix (i.e., ~1 cm^3^ nominal voxel size) during spectral processing steps. Thirty‐two acquisition weighted averages and two‐step phase cycling were applied resulting in a scan time of 15:09 min. Advanced Siemens shimming procedure with manual adjustments was conducted prior to each MRSI scan.

### 2.3 MRSI data analysis

MRSI data was analyzed with a combination of MATLAB (R2013a, MathWorks, Natick, MA), Bash (4.2.25, Free Software Foundation, Boston, MA), MINC (2.0, MINC Tools, McConnell Brain Imaging Center, Montreal, QC, Canada), and LCModel software (6.3‐1, S. Provencher, LCModel, Oakville, ON, Canada). The GAMMA library was used for the creation of two different basis sets, one for the nonedited spectra (containing 21 brain metabolites, including tCr) and one for the difference spectrum (containing GABA+ and Glx among others) (Hnilicova et al., 2016). Cramér–Rao lower bounds (CRLB) thresholds were set at 30% for the quantification of all spectra within the VOI (see Table S1 for mean CRLB values). An ROI‐based quantification (described in Spurny et al., 2019) was applied for the analysis of GABA+ and Glx ratios to total creatine (GABA+/tCr and Glx/tCr), as well as GABA+/Glx ratios in the hippocampus, insula, putamen, pallidum, and thalamus. In short, masks of each ROI were derived from the automated segmentation of structural images using FreeSurfer. Maps of GABA+, Glx and tCr were interpolated to the resolution of structural images (288 × 288 × 208) and overlaid with the derived masks. Mean ratios of GABA+/tCr, Glx/tCr, and GABA+/Glx were calculated for each voxel. Zeros were filtered from the maps and average concentration were calculated within each ROI. ROIs with <90% valid voxels, due to CRLB thresholds, were excluded from subsequent analyses.

### 2.4 Meteorological data

Spindelegger et al. reported a potential link between global radiation and the serotonergic system (Spindelegger et al., 2012). Hence, our analyses were performed with and without measures for accumulated daylight and global radiation to investigate their influence on GABA and glutamate levels. Daily meteorological data from the years 2017–2020 were provided by the Central Institute for Meteorology and Geodynamics in Vienna, Austria (ZAMG, http://www.zamg.ac.at) for the location of Hohe Warte in Vienna. Both the duration of daily sunshine (in hours) and global radiation, defined as the combination of direct solar radiation and diffuse sky radiation received from a unit of surface (in J/cm^2^) were included as covariates into the statistical models. We calculated the accumulated amount of daily sunshine and global radiation for each participant of the last 3, 5, 7, 15, 30, and 90 days prior to their MRSI measurement.

### 2.5 Statistical analyses

Statistical analyses were conducted using SPSS Statistics (v24.0, 2010, SPSS, Inc., an IBM Company, Chicago, IL) and MATLAB. We used different approaches to test for differences between seasons, seasonal patterns in our data set, stationarity (if no seasonal patterns could be found) and associations between neurotransmitter ratios and light exposure. All statistical analyses were corrected for multiple testing using the Sidak correction. However, due to the high number of statistical tests, uncorrected significant *p*‐values <0.01 are presented for exploratory purposes.

#### 2.5.1 Investigation of seasonal differences

In a first step we investigated differences in GABA+/tCr, GABA+/Glx, and Glx/tCr ratios between different time periods using analyses of covariance (ANCOVAs). We tested for differences between the warm and cold period, for seasonal differences, based on meteorological seasons and performed a monthly comparison (see Table S2 for detailed group sizes). Each analysis was conducted including all ROIs in a combined model and additionally for each ROI independently. Sex and age were included as covariates in each model. Additionally, ANCOVAs were run similar to (Spindelegger et al., 2012) with and without accumulated daily sunshine and global radiation of the last 3, 5, 7, 15, 30, and 90 days of Vienna, respectively, as covariate.

#### 2.5.2 Analyses of seasonal patterns using curve fitting models

To test for complex seasonal patterns, polynomial functions up to the 4th degree were fitted using the curve fitting toolbox in Matlab. GABA+/tCr, GABA+/Glx, and Glx/tCr, respectively, of each ROI on the *y*‐axis and day of the year (DOY) on the *x*‐axis were used as input parameters. Sum of squared errors (SSE) and adjusted *R* ^2^ were calculated to evaluate the quality of each fit.

#### 2.5.3 Unit root and stationarity tests

In addition, we tested the data for unit roots and stationarity applying the augmented Dickey‐Fuller test, using two lags (Dickey & Fuller, 1979) in combination with the Kwiatkowski–Phillips–Schmidt–Shin (KPSS) test (Kwiatkowski et al., 1992). KPSS tests were performed for constant + trend specification using the following formula:

$$\text{KPSS}=\frac{\sum_{t=1}^{N}S_{t}^{2}}{N^{2}\lambda^{2}},$$

where *S* is the squared cumulative residual, *λ* is the standard error, and *N* is the number of observations.

Although both tests are designed for longitudinal data, they allow insights into seasonal patterns by providing information on cumulative constant + trend specific residuals in cross‐sectional analyses.

#### 2.5.4 Associations of sun exposure and neurotransmitter ratios

Finally, to test for potential correlations between sun exposure and neurotransmitter ratios, Spearman correlations between GABA+/tCr, GABA+/Glx, and Glx/tCr ratios of each ROI, cumulated daylight (in hours) and global radiation (in J/cm^3^) of the last 3, 5, 7, 15, 30, and 90 days prior to each MRSI scan, respectively, were estimated using SPSS.

## 3 RESULTS

**FIGURE 1:**
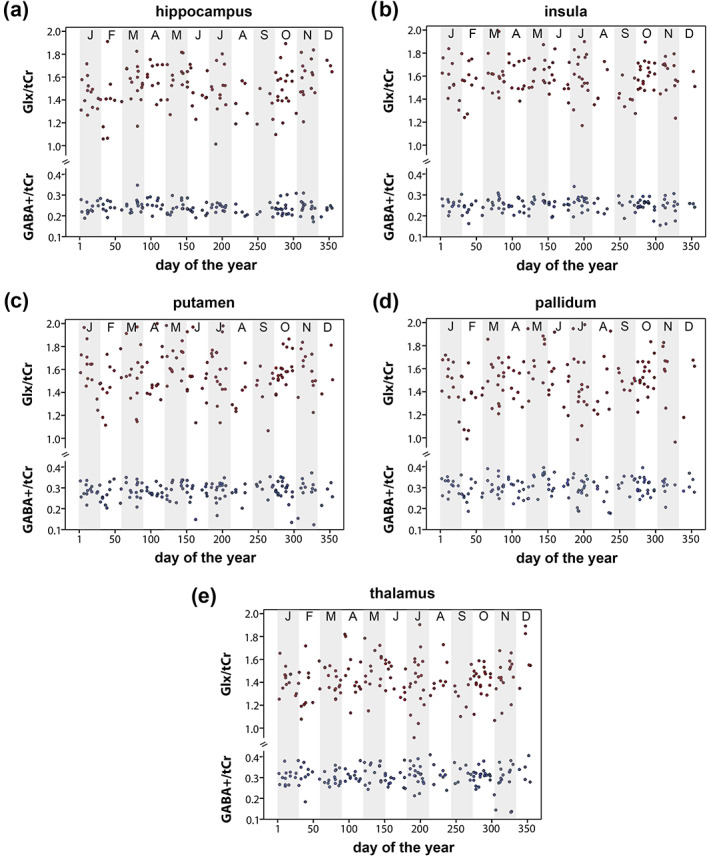
Distribution of GABA+/tCr (blue) and Glx/tCr (red) ratios across the year within the hippocampus (a), insula (b), putamen (c), pallidum (d), and thalamus (e). Gray and white columns represent different months. GABA+, GABA+ macromolecules; Glx, glutamate + glutamine; tCr, total creatine

Data distributions of GABA+/tCr and Glx/tCr within each ROI are presented in Figure 1.

### 3.1 Investigation of seasonal differences

**FIGURE 2:**
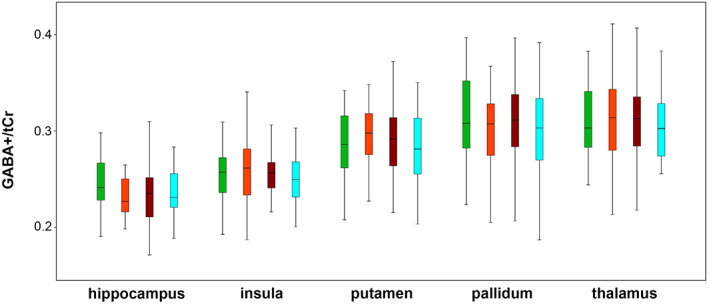
Seasonal concentrations of GABA+/tCr ratios. Boxplots of mean seasonal GABA+/tCr ratios (spring, green; summer, orange; autumn, brown; winter, blue) are depicted for each region investigated. GABA+, GABA+ macromolecules; tCr, total creatine

**FIGURE 3:**
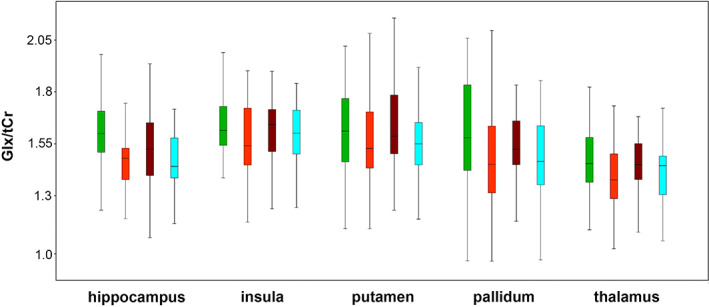
Seasonal concentrations of Glx/tCr ratios. Boxplots of mean seasonal Glx/tCr ratios (spring, green; summer, orange; autumn, brown; winter, blue) are depicted for each region investigated. Glx, glutamate + glutamine; tCr, total creatine

No significant differences were found between warm (April–September) and cold (October–March) periods or seasons (see Figures 2 and 3) in models combining all ROIs and when ROIs were investigated independently, with and without covariates, for each region or neurotransmitter ratio. In a monthly comparison, again no interaction effects were be shown in the models including all ROIs. When ROIs were investigated independently, uncorrected significant differences for Glx/tCr ratios in the hippocampus including covariates of different length of accumulated daylight and global radiation (p_uncorr._ ranging from 0.008 to 0.02) as well as for GABA+/tCr and Glx/tCr ratios in the pallidum (p_uncorr._ ranging from 0.001 to 0.02) were calculated. However, these results did not survive correction for multiple testing and therefore need to be considered as potential false positives.

### 3.2 Analyses of seasonal patterns using curve fitting models

In the next step we tested derived data for more complex patterns using curve fitting functions. No polynomial function could be fitted in the different neurotransmitter data sets of each ROI with an adjusted *R* ^2^ > 0.1. Summed squared errors (SSE) varied between 0.1 and 11.19.

### 3.3 Unit root and stationarity tests

Since our analyses revealed no significant seasonal patterns, we aimed to analyze the neurotransmitter distributions for stationarity across the year utilizing Dickey–Fuller and KPSS statistics. All *t*‐stats derived from the augmented Dickey–Fuller tests exceeded the critical value leading to significant results and thereby suggesting no unit root in the observed data. Moreover, the KPSS test revealed non‐significant results except for hippocampal Glx/tCr ratios (values below both critical value thresholds of 95% and 99%), suggesting stationarity in the data sets of all other neurotransmitter ratios within each ROI (see Table 1).

**TABLE 1:** Derived *t* stats from the augmented Dickey–Fuller test and KPSS statistical values of all neurotransmitter ratios within each investigated brain region

		Augmented Dickey–Fuller statistic critical value (1%) = −3.96	KPSS statistic critical value (99%) = 0.216
Hippocampus	GABA+/tCr	−9.38	0.082
	GABA+/Glx	−9.67	0.069
	Glx/tCr	−10.95	0.219*
Insula	GABA+/tCr	−10.68	0.089
	GABA+/Glx	−10.86	0.129
	Glx/tCr	−10.04	0.118
Putamen	GABA+/tCr	−7.44	0.088
	GABA+/Glx	−6.24	0.160
	Glx/tCr	−10.94	0.120
Pallidum	GABA+/tCr	−9.37	0.050
	GABA+/Glx	−9.32	0.045
	Glx/tCr	−11.56	0.167
Thalamus	GABA+/tCr	−7.75	0.033
	GABA+/Glx	−7.18	0.064
	Glx/tCr	−10.35	0.153

* KPSS statistical value.

Abbreviations: GABA+, GABA+ macromolecules; Glx, glutamate + glutamine; tCr, total creatine.

For hippocampal Glx/tCr ratios, no unit root could be found using the Dickey–Fuller test, while stationarity could not be confirmed by the KPSS test (KPSS statistics of 0.219 minimally exceeding the critical value [99%] of 0.216). Moreover, constant + trend cumulative residuals of the data set may suggest but does not confirm a slight seasonal trend within Glx/tCr ratios of the hippocampus (see Figure S2). Although no seasonal effects could be found in our data distribution, stationarity within this data set could not be ultimately confirmed and results need to be interpreted with caution.

### 3.4 Associations of sun exposure and neurotransmitter ratios

Finally, in our correlation analyses investigating the coherence of sun exposure utilizing cumulated daylight and global radiation of different time periods prior to the measurement day, no significant correlations could be found.

## 4 DISCUSSION

Seasonal adaptions in the dopaminergic and serotonergic neurotransmitter system were frequently shown in previous studies (Eisenberg et al., 2010; Spies et al., 2018; Spindelegger et al., 2012). Variations in the serotonergic system have already been linked to seasonal affective disorders, characterized by depressed mood mainly during autumn and winter (Spies et al., 2018). However, also changes of neurotransmitter systems beside dopamine and serotonin, including the GABAergic and glutamatergic system, were shown to attribute to the pathophysiology of depression (Sanacora et al., 2012). Therefore, we aimed to investigate seasonal variations within MRSI‐derived measures of GABA+ and Glx in healthy participants. Statistical analyses revealed no significant differences between not only the warm and cold period, seasons but also monthly comparisons of GABA+/tCr, Glx/tCr, and GABA+/Glx levels. Moreover, curve fitting approaches did not show fits of seasonal patterns with adequate SSEs. In turn stationarity tests confirmed the absence of seasonal variations in neurotransmitter ratios in our data sets containing baseline neurotransmitter levels in five different subcortical brain regions of healthy individuals. Solely for Glx/tCr ratios of the hippocampus, confirmation of stationarity was not possible to estimate. Hence, stationarity or potential seasonal patterns of Glx levels in the hippocampus should be confirmed in an independent data set in future approaches.

Previous research has mainly focused on the role of GABA and glutamate in seasonal and circadian control within the RHT and SCN, but has neglected their role for seasonal encoding in other brain areas. While it was reported that GABA‐mediated cortical inhibition follows circadian patterns (Lang et al., 2011), effects on other subcortical regions are questionable. Within our study, no significant variations throughout the year of GABA+/tCr, Glx/tCr, or GABA+/Glx measures of the hippocampus, insula, putamen, pallidum, and thalamus could be shown. Although seasonal variations in hippocampal GABA concentrations could be reported in rats (Li et al., 2020), this could not be translated to our human data set. Thus, it can be speculated that potential seasonal influences in the GABAergic or glutamatergic system are not reflected in changes GABA+/tCr and Glx/tCr ratios in downstream areas or are too subtle to be measured using an MRSI approach. Hence, it can be suggested that seasonal effects on GABAergic and glutamatergic neurotransmission might be attributed to the receptor side or solely affect glutamate and not Glx and are therefore not reflected in altered concentrations of total neurotransmitter content (Rohr et al., 2019). Particularly the GABAergic system in the SCN shows a unique behavior. GABA is able to act both in inhibitory and excitatory ways in a reciprocal behavior and thereby increase firing in some neurons, while reducing firing rates in others. Thus, seasonal influences may not lead to changes in the concentration of GABA rather than environmental conditions being able to shape the cellular response to GABA of neurons in the SCN and downstream areas (DeWoskin et al., 2015). However, the SCN itself comprises a brain area, too small to be reliably quantified using MRS techniques, only allowing speculations on neurotransmitter concentrations within this brain region.

Despite variations between different times of the year, the correlation analyses performed in the course of this study did not show any relationship of GABA+/tCr, Glx/tCr, or GABA+/Glx and accumulated daylight or radiation of different periods in any ROI investigated. Other neurotransmitter systems were shown to be involved in light adaption, for example, dopamine as a main factor in retinal function linked to circadian rhythmicity (Witkovsky, 2004). Moreover, studies reported light‐dependent variations of the serotonin 1A receptor (Spindelegger et al., 2012), or patients suffering from SAD, showing reduced levels of light sensitivity (Hebert et al., 2004). However, no associations of daylight and levels of GABA+ or Glx could be shown in our study, suggesting no direct influence of light exposure on total neurotransmitter concentrations. Nevertheless, it has to be stated that no data on individual light exposure of participants was available for our analyses.

Several studies have provided evidence of potential influences on MRS‐derived measures of neurotransmitter concentrations. While a correction of GABA and Glx ratios for age (Gao et al., 2013; Maes et al., 2018) and gender (Spurny‐Dworak et al., 2022) is highly recommended in unbalanced groups, stationarity in our data sets suggests no need for correction of the time of the year for GABA+/tCr, Glx/tCr, or GABA+/Glx ratios of healthy individuals. Since our analyses suggested neurotransmitter ratios to be unaffected by changing heliophysical conditions regarding daylight and temperature throughout the year in healthy participants, seasonal effects on GABA and glutamate levels in patients suffering from SAD, showing changes in light sensitivity (Hebert et al., 2004) will be of high interest in future approaches.

### 4.1 Limitations

While this study benefits from a very large sample size including baseline measures of 159 individuals, some limitations need to be mentioned. The quantification of GABA+ (GABA+ macromolecules, mainly containing lipids and methyl and methylene resonances of proteins; Behar & Ogino, 1993; Povazan et al., 2015) and the combined measure of glutamate and glutamine does not allow analyses of pure GABA and glutamate levels. Moreover, spill‐over effects of adjacent voxels can potentially influence the derived signal in the investigated ROIs. Although we received detailed data on total light exposure and radiation by the ZAMG for each day in the respective time frame, no data on individual light exposure and lifestyle of study participants was available for our analyses. Rather big voxel sizes used in MRSI approaches do not allow quantification of neurotransmitter levels in small brain regions including the SCN. Finally, stationarity tests should be interpreted with caution, since they were used on cross‐sectional and not classical time series data, limiting their validity. Nevertheless, they provide valuable insights into potential seasonal patterns by reporting cumulative constant plus trend specific residuals among other output parameters. Hence, future studies should aim to include longitudinal data of study participants.

## 5 CONCLUSION AND OUTLOOK

Here we show stable patterns of GABA+/tCr, Glx/tCr, and GABA+/Glx levels in five subcortical brain regions, over the course of the year. Despite the important correction for age (Gao et al., 2013; Maes et al., 2018) and gender (Spurny‐Dworak et al., 2022) for unbalanced study groups, when analyzing MRS derived measures of GABA and glutamate, a correction for seasonality does not seem necessary for the subcortical brain regions quantified in the scope of our study. Future approaches should aim to investigate seasonal patterns of neurotransmitter concentrations in disease‐dependent states, especially in seasonal affective disorder.

## CONFLICT OF INTEREST STATEMENT

R. Lanzenberger received investigator‐initiated research funding from Siemens Healthcare regarding clinical research using PET/MR. He is a shareholder of the start‐up company BM Health GmbH since 2019. T. Vanicek recieved speaker honoraria from Janssen. M. Spies received speaker honoraria from Janssen and Austroplant as well as travel grants and/or workshop participation from Janssen, Austroplant, AOP Orphan Pharmaceuticals, and Eli Lilly. The author(s) declared no potential conflicts of interest with respect to the research, authorship, and/or publication of this article.

## Supporting information


**Figure S1.** Exemplary spectrum from the putamen (a) and position of the volume of interest (white) and field of view (yellow) in coronal (b) and horizontal (c) view. GABA+, GABA+ macromolecules; Glx, glutamate + glutamine; tCr, total creatine.

**Figure S2.** Distribution of constant + trend cumulative residuals derived from the KPSS test of hippocampal Glx/tCr ratios. Gray and white columns represent different months (January (J)–December (D)). Glx, glutamate + glutamine; tCr, total creatine.

**Table S1.** Cramér–Rao lower bounds (CRLB) values for each region and metabolite (presented as mean ± standard deviation [minimum–maximum]). GABA+, GABA+ macromolecules; Glx, glutamate + glutamine; tCr, total creatine.

**Table S2.** Detailed sample size for each comparison (warm and cold periods, seasonal and monthly comparisons) within each group and neurotransmitter ratio. GABA+, GABA+ macromolecules; Glx, glutamate + glutamine; tCr, total creatine.

## Data Availability

Due to data protection laws processed data is available from the authors upon reasonable request. Please contact rupert.lanzenberger@meduniwien.ac.at with any questions or requests.

## References

[hbm26236-bib-0001] Alamilla, J. , Granados‐Fuentes, D. , & Aguilar‐Roblero, R. (2015). The anterior paraventricular thalamus modulates neuronal excitability in the suprachiasmatic nuclei of the rat. European Journal of Neuroscience, 42, 2833–2842. 10.1111/ejn.13088 26417679 PMC4737286

[hbm26236-bib-0002] Albers, H. E. , Walton, J. C. , Gamble, K. L. , McNeill, J. K. , & Hummer, D. L. (2017). The dynamics of GABA signaling: Revelations from the circadian pacemaker in the suprachiasmatic nucleus. Frontiers in Neuroendocrinology, 44, 35–82. 10.1016/j.yfrne.2016.11.003 27894927 PMC5225159

[hbm26236-bib-0003] Aumann, T. D. , Raabus, M. , Tomas, D. , Prijanto, A. , Churilov, L. , Spitzer, N. C. , & Horne, M. K. (2016). Differences in number of midbrain dopamine neurons associated with summer and winter photoperiods in humans. PLoS One, 11, e0158847. 10.1371/journal.pone.0158847 27428306 PMC4948786

[hbm26236-bib-0004] Behar, K. L. , & Ogino, T. (1993). Characterization of macromolecule resonances in the 1H NMR spectrum of rat brain. Magnetic Resonance in Medicine, 30, 38–44. 10.1002/mrm.1910300107 8371672

[hbm26236-bib-0005] Bogner, W. , Gagoski, B. , Hess, A. T. , Bhat, H. , Tisdall, M. D. , van der Kouwe, A. J. , Strasser, B. , Marjanska, M. , Trattnig, S. , Grant, E. , Rosen, B. , & Andronesi, O. C. (2014). 3D GABA imaging with real‐time motion correction, shim update and reacquisition of adiabatic spiral MRSI. NeuroImage, 103, 290–302. 10.1016/j.neuroimage.2014.09.032 25255945 PMC4312209

[hbm26236-bib-0006] Bogner, W. , Hess, A. T. , Gagoski, B. , Tisdall, M. D. , van der Kouwe, A. J. , Trattnig, S. , Rosen, B. , & Andronesi, O. C. (2014). Real‐time motion‐ and B0‐correction for LASER‐localized spiral‐accelerated 3D‐MRSI of the brain at 3T. NeuroImage, 88, 22–31. 10.1016/j.neuroimage.2013.09.034 24201013 PMC4010560

[hbm26236-bib-0007] Book, G. A. , Meda, S. A. , Janssen, R. , Dager, A. D. , Poppe, A. , Stevens, M. C. , Assaf, M. , Glahn, D. , & Pearlson, G. D. (2021). Effects of weather and season on human brain volume. PLoS One, 16, e0236303. 10.1371/journal.pone.0236303 33760826 PMC7990212

[hbm26236-bib-0008] Daut, R. A. , & Fonken, L. K. (2019). Circadian regulation of depression: A role for serotonin. Frontiers in Neuroendocrinology, 54, 100746. 10.1016/j.yfrne.2019.04.003 31002895 PMC9826732

[hbm26236-bib-0009] DeWoskin, D. , Myung, J. , Belle, M. D. , Piggins, H. D. , Takumi, T. , & Forger, D. B. (2015). Distinct roles for GABA across multiple timescales in mammalian circadian timekeeping. Proceedings of the National Academy of Sciences of the United States of America, 112, E3911–E3919. 10.1073/pnas.1420753112 26130805 PMC4517259

[hbm26236-bib-0010] Dickey, D. A. , & Fuller, W. A. (1979). Distribution of the estimators for autoregressive time series with a unit root. Journal of the American Statistical Association, 74, 427–431. 10.2307/2286348

[hbm26236-bib-0011] Ebling, F. J. (1996). The role of glutamate in the photic regulation of the suprachiasmatic nucleus. Progress in Neurobiology, 50, 109–132. 10.1016/s0301-0082(96)00032-9 8971980

[hbm26236-bib-0012] Eisenberg, D. P. , Kohn, P. D. , Baller, E. B. , Bronstein, J. A. , Masdeu, J. C. , & Berman, K. F. (2010). Seasonal effects on human striatal presynaptic dopamine synthesis. Journal of Neuroscience, 30, 14691–14694. 10.1523/JNEUROSCI.1953-10.2010 21048126 PMC3010858

[hbm26236-bib-0013] Farajnia, S. , van Westering, T. L. , Meijer, J. H. , & Michel, S. (2014). Seasonal induction of GABAergic excitation in the central mammalian clock. Proceedings of the National Academy of Sciences of the United States of America, 111, 9627–9632. 10.1073/pnas.1319820111 24979761 PMC4084452

[hbm26236-bib-0014] Gao, F. , Edden, R. A. , Li, M. , Puts, N. A. , Wang, G. , Liu, C. , Zhao, B. , Wang, H. , Bai, X. , Zhao, C. , Wang, X. , & Barker, P. B. (2013). Edited magnetic resonance spectroscopy detects an age‐related decline in brain GABA levels. NeuroImage, 78, 75–82. 10.1016/j.neuroimage.2013.04.012 23587685 PMC3716005

[hbm26236-bib-0015] Hannibal, J. (2006). Roles of PACAP‐containing retinal ganglion cells in circadian timing. International Review of Cytology, 251, 1–39. 10.1016/S0074-7696(06)51001-0 16939776

[hbm26236-bib-0016] Hannibal, J. (2021). Comparative neurology of circadian photoreception: The retinohypothalamic tract (RHT) in sighted and naturally blind mammals. Frontiers in Neuroscience, 15, 640113. 10.3389/fnins.2021.640113 34054403 PMC8160255

[hbm26236-bib-0017] Hebert, M. , Beattie, C. W. , Tam, E. M. , Yatham, L. N. , & Lam, R. W. (2004). Electroretinography in patients with winter seasonal affective disorder. Psychiatry Research, 127, 27–34. 10.1016/j.psychres.2004.03.006 15261702

[hbm26236-bib-0018] Hnilicova, P. , Povazan, M. , Strasser, B. , Andronesi, O. C. , Gajdosik, M. , Dydak, U. , Ukropec, J. , Dobrota, D. , Trattnig, S. , & Bogner, W. (2016). Spatial variability and reproducibility of GABA‐edited MEGA‐LASER 3D‐MRSI in the brain at 3 T. NMR in Biomedicine, 29, 1656–1665. 10.1002/nbm.3613 27717093 PMC5095789

[hbm26236-bib-0019] Kaasinen, V. , Jokinen, P. , Joutsa, J. , Eskola, O. , & Rinne, J. O. (2012). Seasonality of striatal dopamine synthesis capacity in Parkinson's disease. Neuroscience Letters, 530, 80–84. 10.1016/j.neulet.2012.09.047 23041046

[hbm26236-bib-0020] Kalueff, A. V. , & Nutt, D. J. (2007). Role of GABA in anxiety and depression. Depression and Anxiety, 24, 495–517. 10.1002/da.20262 17117412

[hbm26236-bib-0021] Kasper, S. , Wehr, T. A. , Bartko, J. J. , Gaist, P. A. , & Rosenthal, N. E. (1989). Epidemiological findings of seasonal changes in mood and behavior. A telephone survey of Montgomery County, Maryland. Archives of General Psychiatry, 46, 823–833. 10.1001/archpsyc.1989.01810090065010 2789026

[hbm26236-bib-0022] Kwiatkowski, D. , Phillips, P. C. B. , Schmidt, P. , & Shin, Y. (1992). Testing the null hypothesis of stationarity against the alternative of a unit root. Journal of Econometrics, 54, 159–178. 10.1016/0304-4076(92)90104-Y

[hbm26236-bib-0023] Lang, N. , Rothkegel, H. , Reiber, H. , Hasan, A. , Sueske, E. , Tergau, F. , Ehrenreich, H. , Wuttke, W. , & Paulus, W. (2011). Circadian modulation of GABA‐mediated cortical inhibition. Cerebral Cortex, 21, 2299–2306. 10.1093/cercor/bhr003 21350047

[hbm26236-bib-0024] Li, T. , Wang, H. , Zhang, H. , Liu, L. , Li, P. , & Ma, S. (2020). Effect of the pineal gland on 5‐hydroxytryptamine and γ‐aminobutyric acid secretion in the hippocampus of male rats during the summer and winter. Journal of Traditional Chinese Medical Sciences, 7, 283–290. 10.1016/j.jtcms.2020.07.004

[hbm26236-bib-0025] Maes, C. , Hermans, L. , Pauwels, L. , Chalavi, S. , Leunissen, I. , Levin, O. , Cuypers, K. , Peeters, R. , Sunaert, S. , Mantini, D. , Puts, N. A. J. , Edden, R. A. E. , & Swinnen, S. P. (2018). Age‐related differences in GABA levels are driven by bulk tissue changes. Human Brain Mapping, 39, 3652–3662. 10.1002/hbm.24201 29722142 PMC6866434

[hbm26236-bib-0026] Mc Mahon, B. , Andersen, S. B. , Madsen, M. K. , Hjordt, L. V. , Hageman, I. , Dam, H. , Svarer, C. , da Cunha‐Bang, S. , Baare, W. , Madsen, J. , Hasholt, L. , Holst, K. , Frokjaer, V. G. , & Knudsen, G. M. (2016). Seasonal difference in brain serotonin transporter binding predicts symptom severity in patients with seasonal affective disorder. Brain, 139, 1605–1614. 10.1093/brain/aww043 26994750

[hbm26236-bib-0027] Mendoza, J. (2017). Circadian neurons in the lateral habenula: Clocking motivated behaviors. Pharmacology, Biochemistry, and Behavior, 162, 55–61. 10.1016/j.pbb.2017.06.013 28666896

[hbm26236-bib-0028] Meyer, C. , Muto, V. , Jaspar, M. , Kusse, C. , Lambot, E. , Chellappa, S. L. , Degueldre, C. , Balteau, E. , Luxen, A. , Middleton, B. , Archer, S. N. , Collette, F. , Dijk, D. J. , Phillips, C. , Maquet, P. , & Vandewalle, G. (2016). Seasonality in human cognitive brain responses. Proceedings of the National Academy of Sciences of the United States of America, 113, 3066–3071. 10.1073/pnas.1518129113 26858432 PMC4801294

[hbm26236-bib-0029] Myung, J. , Hong, S. , DeWoskin, D. , De Schutter, E. , Forger, D. B. , & Takumi, T. (2015). GABA‐mediated repulsive coupling between circadian clock neurons in the SCN encodes seasonal time. Proceedings of the National Academy of Sciences of the United States of America, 112, E3920–E3929. 10.1073/pnas.1421200112 26130804 PMC4517217

[hbm26236-bib-0030] Norgaard, M. , Ganz, M. , Svarer, C. , Fisher, P. M. , Churchill, N. W. , Beliveau, V. , Grady, C. , Strother, S. C. , & Knudsen, G. M. (2017). Brain networks implicated in seasonal affective disorder: A neuroimaging PET study of the serotonin transporter. Frontiers in Neuroscience, 11, 614. 10.3389/fnins.2017.00614 29163018 PMC5682039

[hbm26236-bib-0031] Ono, D. , Honma, K. I. , & Honma, S. (2021). GABAergic mechanisms in the suprachiasmatic nucleus that influence circadian rhythm. Journal of Neurochemistry, 157, 31–41. 10.1111/jnc.15012 32198942

[hbm26236-bib-0032] Partonen, T. , & Lonnqvist, J. (1998). Seasonal affective disorder. Lancet, 352, 1369–1374. 10.1016/S0140-6736(98)01015-0 9802288

[hbm26236-bib-0033] Pastrnak, M. , Simkova, E. , & Novak, T. (2021). Insula activity in resting‐state differentiates bipolar from unipolar depression: A systematic review and meta‐analysis. Scientific Reports, 11, 16930. 10.1038/s41598-021-96319-2 34417487 PMC8379217

[hbm26236-bib-0034] Pjrek, E. , Friedrich, M. E. , Cambioli, L. , Dold, M. , Jager, F. , Komorowski, A. , Lanzenberger, R. , Kasper, S. , & Winkler, D. (2020). The efficacy of light therapy in the treatment of seasonal affective disorder: A meta‐analysis of randomized controlled trials. Psychotherapy and Psychosomatics, 89, 17–24. 10.1159/000502891 31574513

[hbm26236-bib-0035] Porcu, A. , Nilsson, A. , Booreddy, S. , Barnes, S. A. , Welsh, D. K. , & Dulcis, D. (2022). Seasonal changes in day length induce multisynaptic neurotransmitter switching to regulate hypothalamic network activity and behavior. Science Advances, 8, eabn9867. 10.1126/sciadv.abn9867 36054362 PMC10848959

[hbm26236-bib-0036] Povazan, M. , Hangel, G. , Strasser, B. , Gruber, S. , Chmelik, M. , Trattnig, S. , & Bogner, W. (2015). Mapping of brain macromolecules and their use for spectral processing of (1)H‐MRSI data with an ultra‐short acquisition delay at 7 T. NeuroImage, 121, 126–135. 10.1016/j.neuroimage.2015.07.042 26210813

[hbm26236-bib-0037] Praschak‐Rieder, N. , Willeit, M. , Wilson, A. A. , Houle, S. , & Meyer, J. H. (2008). Seasonal variation in human brain serotonin transporter binding. Archives of General Psychiatry, 65, 1072–1078. 10.1001/archpsyc.65.9.1072 18762593

[hbm26236-bib-0038] Rohr, K. E. , Pancholi, H. , Haider, S. , Karow, C. , Modert, D. , Raddatz, N. J. , & Evans, J. (2019). Seasonal plasticity in GABAA signaling is necessary for restoring phase synchrony in the master circadian clock network. eLife, 8, e49578. 10.7554/eLife.49578 31746738 PMC6867713

[hbm26236-bib-0039] Ruby, N. F. , Hwang, C. E. , Wessells, C. , Fernandez, F. , Zhang, P. , Sapolsky, R. , & Heller, H. C. (2008). Hippocampal‐dependent learning requires a functional circadian system. Proceedings of the National Academy of Sciences of the United States of America, 105, 15593–15598. 10.1073/pnas.0808259105 18832172 PMC2563080

[hbm26236-bib-0040] Sacchet, M. D. , Camacho, M. C. , Livermore, E. E. , Thomas, E. A. C. , & Gotlib, I. H. (2017). Accelerated aging of the putamen in patients with major depressive disorder. Journal of Psychiatry & Neuroscience, 42, 164–171. 10.1503/jpn.160010 27749245 PMC5403661

[hbm26236-bib-0041] Sanacora, G. , Treccani, G. , & Popoli, M. (2012). Towards a glutamate hypothesis of depression: An emerging frontier of neuropsychopharmacology for mood disorders. Neuropharmacology, 62, 63–77. 10.1016/j.neuropharm.2011.07.036 21827775 PMC3205453

[hbm26236-bib-0042] Schnellbacher, G. J. , Rajkumar, R. , Veselinovic, T. , Ramkiran, S. , Hagen, J. , Shah, N. J. , & Neuner, I. (2022). Structural alterations of the insula in depression patients—A 7‐Tesla‐MRI study. NeuroImage Clinical, 36, 103249. 10.1016/j.nicl.2022.103249 36451355 PMC9668670

[hbm26236-bib-0043] Schur, R. R. , Draisma, L. W. , Wijnen, J. P. , Boks, M. P. , Koevoets, M. G. , Joels, M. , Klomp, D. W. , Kahn, R. S. , & Vinkers, C. H. (2016). Brain GABA levels across psychiatric disorders: A systematic literature review and meta‐analysis of (1) H‐MRS studies. Human Brain Mapping, 37, 3337–3352. 10.1002/hbm.23244 27145016 PMC6867515

[hbm26236-bib-0044] Silberbauer, L. R. , Spurny, B. , Handschuh, P. , Klobl, M. , Bednarik, P. , Reiter, B. , Ritter, V. , Trost, P. , Konadu, M. E. , Windpassinger, M. , Stimpfl, T. , Bogner, W. , Lanzenberger, R. , & Spies, M. (2020). Effect of ketamine on limbic GABA and glutamate: A human In vivo multivoxel magnetic resonance spectroscopy study. Frontiers in Psychiatry, 11, 549903. 10.3389/fpsyt.2020.549903 33101078 PMC7507577

[hbm26236-bib-0045] Snider, K. H. , Sullivan, K. A. , & Obrietan, K. (2018). Circadian regulation of hippocampal‐dependent memory: Circuits, synapses, and molecular mechanisms. Neural Plasticity, 2018, 7292540. 10.1155/2018/7292540 29593785 PMC5822921

[hbm26236-bib-0046] Spies, M. , James, G. M. , Vraka, C. , Philippe, C. , Hienert, M. , Gryglewski, G. , Komorowski, A. , Kautzky, A. , Silberbauer, L. , Pichler, V. , Kranz, G. S. , Nics, L. , Balber, T. , Baldinger‐Melich, P. , Vanicek, T. , Spurny, B. , Winkler‐Pjrek, E. , Wadsak, W. , Mitterhauser, M. , … Winkler, D. (2018). Brain monoamine oxidase A in seasonal affective disorder and treatment with bright light therapy. Translational Psychiatry, 8, 198. 10.1038/s41398-018-0227-2 30242221 PMC6155094

[hbm26236-bib-0047] Spindelegger, C. , Stein, P. , Wadsak, W. , Fink, M. , Mitterhauser, M. , Moser, U. , Savli, M. , Mien, L. K. , Akimova, E. , Hahn, A. , Willeit, M. , Kletter, K. , Kasper, S. , & Lanzenberger, R. (2012). Light‐dependent alteration of serotonin‐1A receptor binding in cortical and subcortical limbic regions in the human brain. World Journal of Biological Psychiatry, 13, 413–422. 10.3109/15622975.2011.630405 22111663

[hbm26236-bib-0048] Spurny, B. , Heckova, E. , Seiger, R. , Moser, P. , Klobl, M. , Vanicek, T. , Spies, M. , Bogner, W. , & Lanzenberger, R. (2019). Automated ROI‐based labeling for multi‐voxel magnetic resonance spectroscopy data using FreeSurfer. Frontiers in Molecular Neuroscience, 12, 28. 10.3389/fnmol.2019.00028 30837839 PMC6382749

[hbm26236-bib-0049] Spurny, B. , Vanicek, T. , Seiger, R. , Reed, M. B. , Klobl, M. , Ritter, V. , Unterholzner, J. , Godbersen, G. M. , Silberbauer, L. R. , Pacher, D. , Klug, S. , Konadu, M. E. , Gryglewski, G. , Trattnig, S. , Bogner, W. , & Lanzenberger, R. (2021). Effects of SSRI treatment on GABA and glutamate levels in an associative relearning paradigm. NeuroImage, 232, 117913. 10.1016/j.neuroimage.2021.117913 33657450 PMC7610796

[hbm26236-bib-0050] Spurny‐Dworak, B. , Handschuh, P. , Spies, M. , Kaufmann, U. , Seiger, R. , Klobl, M. , Konadu, M. E. , Reed, M. B. , Ritter, V. , Baldinger‐Melich, P. , Bogner, W. , Kranz, G. S. , & Lanzenberger, R. (2022). Effects of sex hormones on brain GABA and glutamate levels in a cis‐ and transgender cohort. Psychoneuroendocrinology, 138, 105683. 10.1016/j.psyneuen.2022.105683 35176535

[hbm26236-bib-0051] Talati, A. , van Dijk, M. T. , Pan, L. , Hao, X. , Wang, Z. , Gameroff, M. , Dong, Z. , Kayser, J. , Shankman, S. , Wickramaratne, P. J. , Posner, J. , & Weissman, M. M. (2022). Putamen structure and function in familial risk for depression: A multimodal imaging study. Biological Psychiatry, 92, 932–941. 10.1016/j.biopsych.2022.06.035 36038379 PMC9872322

[hbm26236-bib-0052] Willeit, M. , Sitte, H. H. , Thierry, N. , Michalek, K. , Praschak‐Rieder, N. , Zill, P. , Winkler, D. , Brannath, W. , Fischer, M. B. , Bondy, B. , Kasper, S. , & Singer, E. A. (2008). Enhanced serotonin transporter function during depression in seasonal affective disorder. Neuropsychopharmacology, 33, 1503–1513. 10.1038/sj.npp.1301560 17882235

[hbm26236-bib-0053] Witkovsky, P. (2004). Dopamine and retinal function. Documenta Ophthalmologica, 108, 17–40. 10.1023/b:doop.0000019487.88486.0a 15104164

