# Supplementary figures and images for: The influence of season on glutamate and GABA levels in the healthy human brain investigated by magnetic resonance spectroscopy imaging
Source: Hum Brain Mapp. 2023 Feb 25;44(6):2654–63. doi: 10.1002/hbm.26236 (PMC10028653; doi:10.1002/hbm.26236)

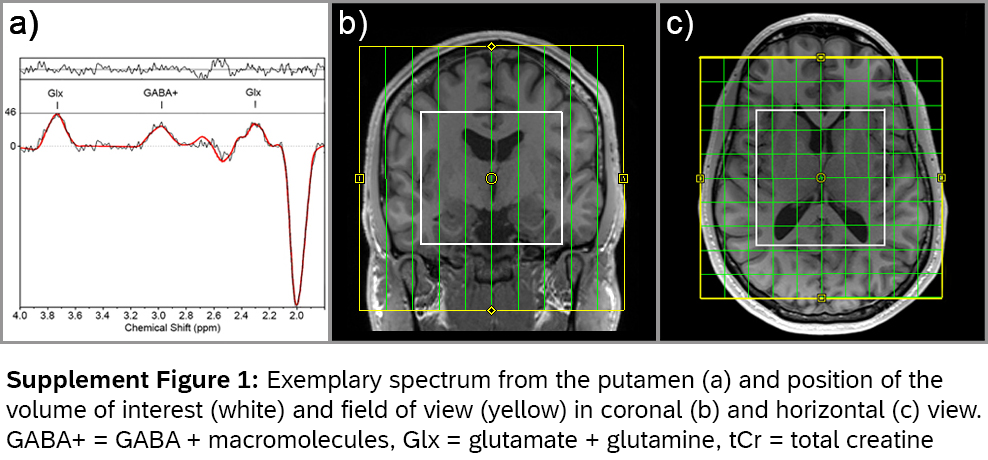

Supplement: Supplementary file 1 — Figure S1. Exemplary spectrum from the putamen (a) and position of the volume of interest (white) and field of view (yellow) in coronal (b) and horizontal (c) view. GABA+, GABA+ macromolecules; Glx, glutamate + glutamine; tCr, total creatine. [file HBM-44-2654-s002.tif]

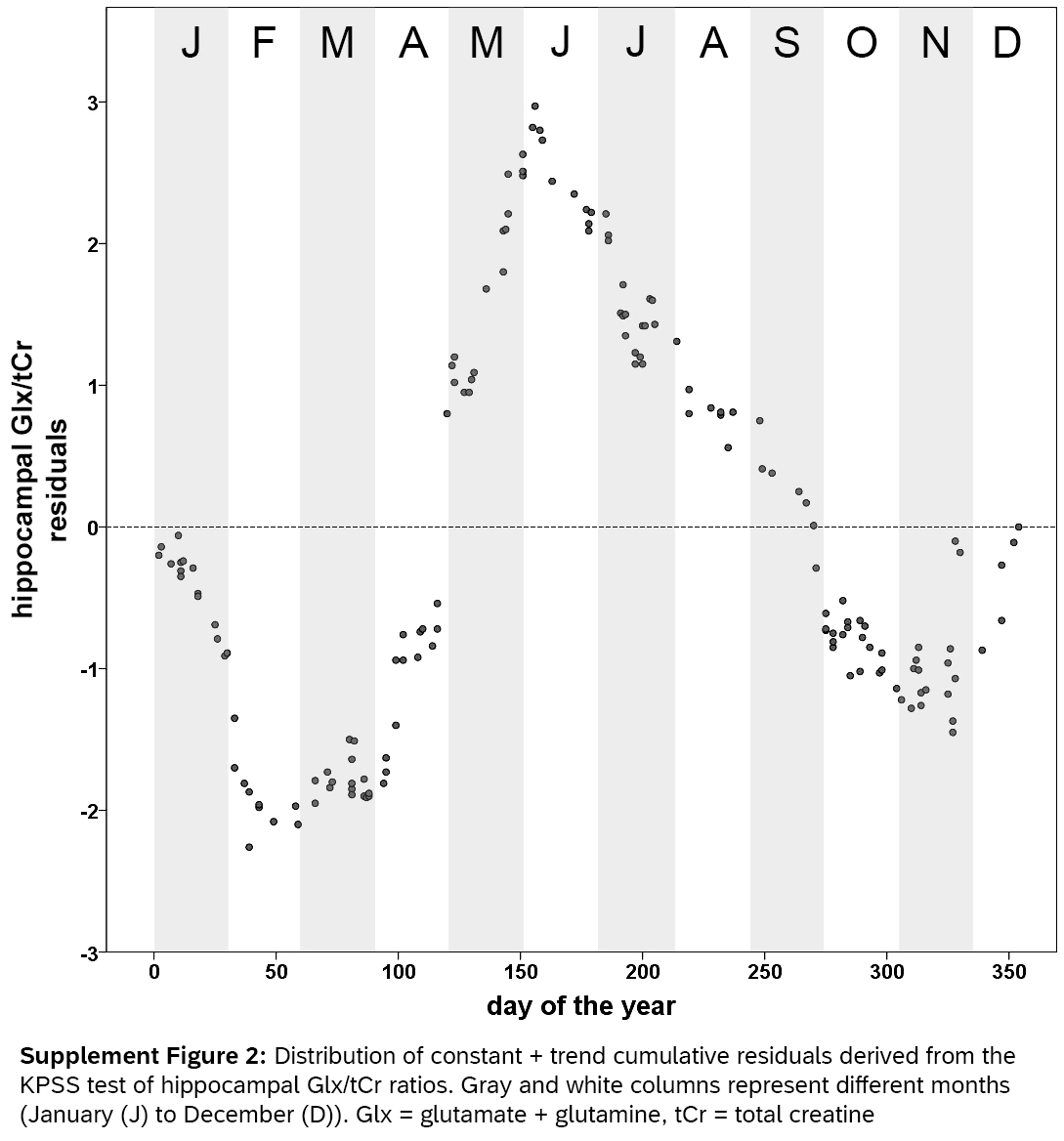

Supplement: Supplementary file 2 — Figure S2. Distribution of constant + trend cumulative residuals derived from the KPSS test of hippocampal Glx/tCr ratios. Gray and white columns represent different months (January (J)–December (D)). Glx, glutamate + glutamine; tCr, total creatine. [file HBM-44-2654-s003.tif]
