# Supplementary material for: The influence of season on glutamate and GABA levels in the healthy human brain investigated by magnetic resonance spectroscopy imaging
Source: Hum Brain Mapp. 2023 Feb 25;44(6):2654–63. doi: 10.1002/hbm.26236 (PMC10028653; doi:10.1002/hbm.26236)
Supplement: Supplementary file 3 — Table S1. Cramér–Rao lower bounds (CRLB) values for each region and metabolite (presented as mean ± standard deviation [minimum–maximum]). GABA+, GABA+ macromolecules; Glx, glutamate + glutamine; tCr, total creatine. [file HBM-44-2654-s004.docx]

|  | **hippocampus** | **insula** | **putamen** | **pallidum** | **thalamus** |
| --- | --- | --- | --- | --- | --- |
| **GABA+ (mean ± SD [min max])** | 16.7 ± 2.4 [9.6-25.9] | 15.9 ± 2.6 [10.6-27.8] | 12.6 ± 2.2 [8.4-20.6] | 12.3 ± 2.4 [7.7-21.1] | 12.0 ± 2.1 [8.1-24.6] |
| **Glx (mean ± SD [min max])** | 10.3 ± 2.6 [5.9-18.3] | 9.7 ± 1.9 [6.3-17.0] | 8.8 ± 1.8 [5.4-15.5] | 9.0 ± 2.0 [5.6-15.5] | 8.2 ± 2.0 [5.4-14.8] |
| **tCr (mean ± SD [min max])** | 5.1 ± 1.6 [2.6-9.9] | 4.9 ± 1.4 [2.8-9.9] | 4.6 ± 1.7 [2.5-9.6] | 4.4 ± 1.4 [2.4-8.5] | 3.2 ± 1.0 [2.1-7.4] |

**Supplement Table 1**: Cramér–Rao lower bounds (CRLB) values for each region and metabolite (presented as mean ± standard deviation [minimum-maximum]). GABA+= GABA + macromolecules, Glx = glutamate + glutamine, tCr = total creatine
