# Supplementary material for: The influence of season on glutamate and GABA levels in the healthy human brain investigated by magnetic resonance spectroscopy imaging
Source: Hum Brain Mapp. 2023 Feb 25;44(6):2654–63. doi: 10.1002/hbm.26236 (PMC10028653; doi:10.1002/hbm.26236)
Supplement: Supplementary file 4 — Table S2. Detailed sample size for each comparison (warm and cold periods, seasonal and monthly comparisons) within each group and neurotransmitter ratio. GABA+, GABA+ macromolecules; Glx, glutamate + glutamine; tCr, total creatine. [file HBM-44-2654-s001.docx]

|  | **hippocampus** | | | **insula** | | | **putamen** | | | **pallidum** | | | **thalamus** | | |
| --- | --- | --- | --- | --- | --- | --- | --- | --- | --- | --- | --- | --- | --- | --- | --- |
|  | **GABA+/tCr** | **Glx/tCr** | **GABA+/Glx** | **GABA+/tCr** | **Glx/tCr** | **GABA+/Glx** | **GABA+/tCr** | **Glx/tCr** | **GABA+/Glx** | **GABA+/tCr** | **Glx/tCr** | **GABA+/Glx** | **GABA+/tCr** | **Glx/tCr** | **GABA+/Glx** |
| warm | 56 | 72 | 56 | 61 | 74 | 61 | 68 | 75 | 68 | 67 | 72 | 67 | 72 | 74 | 72 |
| cold | 76 | 83 | 77 | 71 | 80 | 71 | 76 | 81 | 76 | 72 | 75 | 72 | 80 | 82 | 80 |
| spring | 40 | 46 | 40 | 46 | 46 | 37 | 42 | 42 | 42 | 40 | 44 | 40 | 40 | 45 | 43 |
| summer | 23 | 33 | 23 | 33 | 34 | 29 | 32 | 32 | 32 | 33 | 34 | 33 | 33 | 35 | 35 |
| autumn | 41 | 46 | 41 | 46 | 45 | 38 | 40 | 40 | 40 | 36 | 39 | 36 | 36 | 46 | 44 |
| winter | 28 | 30 | 28 | 30 | 29 | 28 | 30 | 30 | 30 | 30 | 30 | 30 | 30 | 30 | 30 |
| January | 14 | 15 | 14 | 13 | 14 | 13 | 15 | 15 | 15 | 15 | 15 | 15 | 15 | 15 | 15 |
| February | 9 | 10 | 9 | 10 | 10 | 10 | 10 | 10 | 10 | 10 | 10 | 10 | 10 | 10 | 10 |
| March | 15 | 16 | 15 | 14 | 15 | 14 | 15 | 15 | 15 | 15 | 15 | 15 | 15 | 15 | 15 |
| April | 10 | 14 | 10 | 10 | 15 | 10 | 11 | 15 | 11 | 11 | 14 | 11 | 12 | 14 | 12 |
| May | 15 | 16 | 15 | 14 | 15 | 14 | 16 | 16 | 16 | 15 | 15 | 15 | 16 | 16 | 16 |
| June | 9 | 10 | 9 | 9 | 11 | 9 | 10 | 10 | 10 | 9 | 10 | 9 | 10 | 10 | 10 |
| July | 12 | 17 | 12 | 15 | 18 | 15 | 17 | 18 | 17 | 16 | 17 | 16 | 18 | 18 | 18 |
| August | 5 | 8 | 5 | 5 | 7 | 5 | 6 | 8 | 6 | 8 | 8 | 8 | 8 | 8 | 8 |
| September | 5 | 7 | 5 | 8 | 8 | 8 | 8 | 8 | 8 | 8 | 8 | 8 | 8 | 8 | 8 |
| October | 18 | 20 | 18 | 18 | 20 | 18 | 19 | 20 | 19 | 19 | 19 | 19 | 20 | 20 | 20 |
| November | 15 | 17 | 15 | 13 | 17 | 13 | 13 | 16 | 13 | 8 | 11 | 8 | 15 | 17 | 15 |
| December | 5 | 5 | 5 | 3 | 4 | 3 | 4 | 5 | 4 | 5 | 5 | 5 | 5 | 5 | 5 |
| **total number** | **132** | **155** | **132** | **132** | **154** | **132** | **144** | **156** | **144** | **139** | **147** | **139** | **152** | **156** | **152** |

**Supplement Table 2**: Detailed sample size for each comparison (warm & cold periods, seasonal and monthly comparisons) within each group and neurotransmitter ratio. GABA+= GABA + macromolecules, Glx = glutamate + glutamine, tCr = total creatine
